# A rare tumoral combination, synchronous lung adenocarcinoma and mantle cell lymphoma of the pleura

**DOI:** 10.1186/1477-7819-6-137

**Published:** 2008-12-29

**Authors:** Dimitrios Hatzibougias, Mattheos Bobos, Georgia Karayannopoulou, Georgios Karkavelas, Georgios T Karapanagiotidis, Christophoros N Foroulis, Ioannis Kostopoulos

**Affiliations:** 1 Aristotle University of Thessaloniki Medical School, Department of Pathology, Thessaloniki, Greece; 2 Aristotle University of Thessaloniki Medical School, AHEPA University Hospital, Department of Cardio-Thoracic Surgery, Thessaloniki, Greece

## Abstract

**Background:**

Coexistence of adenocarcinoma and mantle cell lymphoma in the same or different anatomical sites is extremely rare. We present a case of incidental discovery of primary lung adenocarcinoma and mantle cell lymphoma involving the pleura, during an axillary thoracotomy performed for a benign condition.

**Case presentation:**

A 73-year old male underwent bullectomy and apical pleurectomy for persistent pneumothorax. A bulla of the lung apex was resected en bloc with a scar-like lesion of the lung, which was located in proximity with the bulla origin, by a wide wedge resection. Histologic examination of the stripped-off parietal pleura and of the bullectomy specimen revealed the synchronous occurrence of two distinct neoplasms, a lymphoma infiltrating the pleura and a primary, early lung adenocarcinoma. Immunohistochemical and fluorescence in situ hybridization assays were performed. The morphologic, immunophenotypic and genetic findings supported the diagnosis of primary lung adenocarcinoma (papillary subtype) coexisting with a non-Hodgkin, B-cell lineage, mantle cell lymphoma involving both, visceral and parietal pleura and without mediastinal lymph node involvement. The neoplastic lymphoid cells showed the characteristic immunophenotype of mantle cell lymphoma and the translocation t(11;14). The patient received 6 cycles of chemotherapy, while pulmonary function tests precluded further pulmonary parenchyma resection (lobectomy) for his adenocarcinoma. The patient is alive and without clinical and radiological findings of local recurrence or distant relapse from both tumors 14 months later.

**Conclusion:**

This is the first reported case of a rare tumoral combination involving simultaneously lung and pleura, emphasizing at the incidental discovery of the two coexisting neoplasms during a procedure performed for a benign condition. Any tissue specimen resected during operations performed for non-tumoral conditions should be routinely sent for pathologic examination.

## Background

Recent epidemiologic evidence suggest that lung cancer is the leading cause of cancer mortality in both sexes and is distinguished according to histopathologic features in two large categories, the small cell lung carcinoma (SCLC) and the non-small cell lung carcinoma (NSCLC) [1]. The most common type of the latter is lung adenocarcinoma which is usually presented as single or multiple poorly circumscribed peripheral lung lesions [2]. Papillary adenocarcinoma is a rare histopathologic subtype of lung adenocarcinoma which is characterized by predominantly papillary structures that replaces the underlying lung parenchyma and has been stated to be associated with poorer prognosis [3,4].

Mantle cell lymphoma (MCL) is a distinct type of B-cell non-Hodgkin lymphoma characterized by t(11;14)(q13;q32) and Cyclin D1 over-expression, comprising from 3% to 10% of all non-Hodgkin's lymphomas [5,6]. The affected patients are mainly middle-aged or older and they are often presented with advanced stage disease (Stages III-IV), frequently involving multiple extranodal sites [7].

Synchronous occurrence of lung adenocarcinoma and malignant lymphoma of the pleura is not reported until today and we report the unique case of a lung adenocarcinoma coexisting with a mantle cell lymphoma of the pleura, which were incidentally discovered during an operation for pneumothorax.

## Case presentation

**Figure 1:**
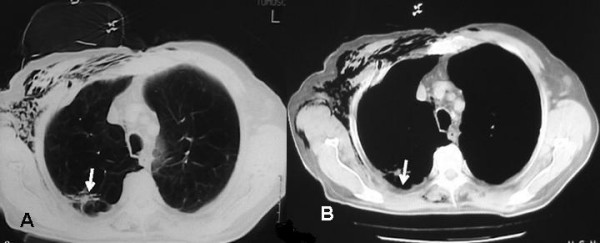
**Preoperative chest CT scan**. (A) Diffuse emphysematous changes of the right upper lobe, surgical subcutaneous emphysema and a scar-like lesion located in the periphery of the posterior segment of the right upper lobe (arrow). (B) Thickening of the parietal pleura in proximity to the scar-like lesion (arrow).

A 73-year old man, heavy smoker, with history of chronic obstructive pulmonary disease, coronary artery disease and HBV infection was admitted to a district hospital because of dyspnea and fever (38–38.5°C). Clinical and radiologic findings revealed spontaneous pneumothorax on the right side, which was initially managed by chest tube drainage. Due to persistent for more than 10 days air leak through the chest tube, he was referred to the department of Cardiothoracic Surgery at AHEPA University Hospital. CT scan imaging of the thorax showed diffuse emphysema, surgical subcutaneous emphysema on the right side as the result of previous chest tube drainage, a scar-like lesion that was located in the periphery of the posterior segment of the right upper lobe (Fig. 1a) and thickening of parietal pleura adjacent to the lung scar (Fig. 1b). Because of the prolonged air leak, the patient underwent apical bullectomy and apical parietal pleura resection to achieve pleurodesis through an axillary thoracotomy. The scar-like lesion of the lung was resected en bloc with the bullectomy specimen by a wide wedge resection of lung parenchyma.

### Pathological findings and immunohistochemistry

On macroscopic examination of the bullectomy specimen, a solid yellowish nodular lesion measuring up to 1.6 cm was found. Close to that lesion many cystic spaces were found.

**Figure 2:**
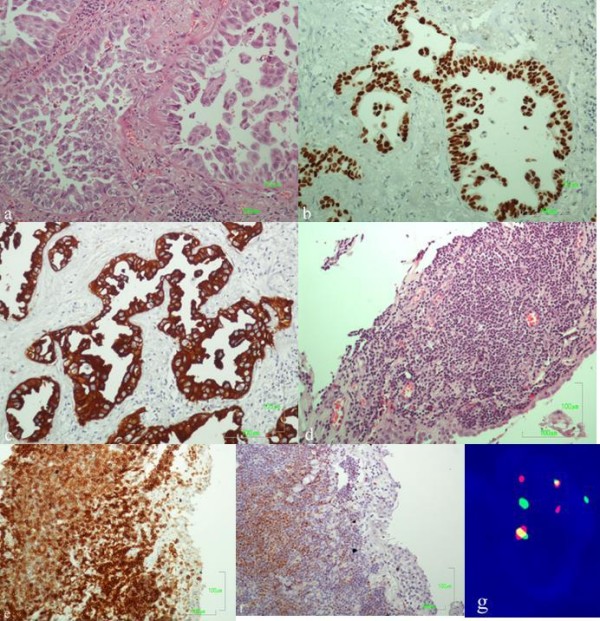
**Lung adenocarcinoma with micropapillary pattern (A, E & H)**. Neoplastic ducts showing strong nuclear staining for TTF-1 (B) and cytokeratin 7 (C). Mantle cell lymphoma with diffuse infiltration of the pleura (D, E & H). Lymphomatous cells, positive for CD5 (E) and cyclin D1 (F). FISH method using the LSI IGH/CCND1 dual color, dual fusion translocation probe: nucleus of neoplastic lymphoid cell with t(11;14) displaying the signal pattern 2 yellow (fusion translocation signals), 2 red (CCND1 gene) and 2 green (IGH gene) (G).

The histologic examination of hematoxylin-eosin-stained sections revealed an adenocarcinoma composed of atypical adenoid formations showing intraluminal papillary projections, some of them detached (Fig. 2a). The tumor cells were tall-cylindrical, with pale eosinophilic cytoplasm and polymorphic nuclei with or without prominent nucleoli. The mitotic activity was low (< 2 mitoses per 10 HPF). There were also foci of necrosis, hyalinosis and ossification. Immunohistochemical stains showed the following tumor-cell immunophenotype: TTF-1(+) (Novocastra, UK), CK7(+) (Dako, DK), CK20(+) (Dako), EGFR(+) (Zymed, USA), p53(+) (Dako), Cyclin D1(+) (Spring, USA), Calretinin(-) (Dako) (Fig. 2b, 2c). The adjacent lung parenchyma showed serious emphysematic changes.

The visceral pleura proximal to the adenocarcinoma and the separately sent to the laboratory specimen of stripped-off parietal pleura tissue showed a band-like diffuse infiltration from neoplastic lymphoid cells (Fig. 2d). The lymphoid cells were medium-sized, with rounded or angular nuclei and with one or more indistinct nucleoli. Focally there was a nodular pattern of growth. The immunophenotype of the lymphoma cells was the following: CD20(+) (Dako), CD45RA(+) (Dako), CD5(+) (Novocastra), Cyclin D1(+) (Spring), CD45RO(-) (Dako), CD3(-) (Novocastra), CD23(-) (Novocastra), EGFR(-), Calretinin(-) (Fig. 2e, 2f). The CD23 immunostain revealed a residual network of dendritic cells, referring to infiltration of preexisting germinal centers.

### Fluorescence in situ hybridization (FISH)

Metaphase FISH analysis was also performed using EGFR/CEP7, CCND1/CEP11 dual color LSI probes and LSI IGH/CCND1 dual color, dual fusion translocation probe (all from Abbott Molecular, USA). Gains of EGFR and CCND1 genes were observed in the adenocarcinoma. On the other hand, in MCL the characteristic translocation between CCND1 gene on chromosome 11 and IGH gene on chromosome 14 [t(11;14)] was found (Fig. 2g). In addition CCND1 gene was amplified, whereas EGFR gene was on normal range.

The morphologic, immunophenotypic and genetic findings supported the diagnosis of primary lung adenocarcinoma (papillary subtype) coexisting with a non-Hodgkin, B-cell lineage, mantle cell lymphoma involving both, visceral and parietal pleura and without mediastinal lymph node involvement.

### Follow-up

The patient received 6 cycles of chemotherapy (Endoxan, Farmorubicin and Vincristine) and his clinical status, 14 months after the diagnosis is good, without any evidence of local recurrence, metastatic disease or lymph node involvement, according to the follow-up CT scan imaging of thorax, abdomen and brain.

## Discussion

Synchronous occurrence of lung adenocarcinoma and mantle cell lymphoma of the pleura is not reported in the medical literature until today. Extensive search of the literature revealed few cases with coexistence of different types of lung carcinomas and malignant lymphomas. Chanel et al. described a synchronous pulmonary adenocarcinoma and extranodal marginal zone lymphoma of MALT type [8]. Rothenburger *et al*. reported a non-Hodgkin's lymphoma coexisting with a NSCLC, whereas Rubiales et al. described the synchronous occurrence of a small-cell lung cancer and a Hodgkin lymphoma [9,10].

Coexistence of adenocarcinoma and mantle cell lymphoma in other anatomical sites, such as the large bowel, has been reported in the past. In total we found four cases concerning colonic involvement. Hopster *et al*, described 2 foci of colonic adenocarcinoma associated with MCL [11]. Kanehira *et al*., presented also 2 cases of invasive adenocarcinoma of the colon coexisting with early MCL [12]. The fourth case reported by Padmanabhan et al, concerned the synchronous presence of adenocarcinoma located in the cecum and MCL involving the colon, the terminal ileum and the regional lymph nodes [13]. Another case of synchronous existence of nodal mantle cell lymphoma and metastatic in mediastinal lymph nodes small cell lung carcinoma was reported by Kampalath et al [14].

Lung adenocarcinoma usually arises from peripheral small bronchi and may be associated with a lung scar (scar adenocarcinoma). It is composed of malignant glandular epithelium which may vary in degree of differentiation from tumor to tumor. Well differentiated tumors may form distinct glands, while others may vary from forming papillary structures to solid tumors without any gland formation. The prognosis depends on the histologic type, clinical stage, and the patient's performance status. A micropapillary pattern is a predictor of poor prognosis [3,15]. The presence of this component should alert the clinician to search more carefully for clinically "silent" metastases.

MCL is a neoplasm of monomorphous small to medium-sized B lymphocytes with irregular nuclei, which resemble the cleaved cells (centrocytes) of germinal centers. Neoplastic transformed cells (centroblasts or immunoblasts) are absent. Tumor cells are typically CD5(+) and CD23(-). The vast majority overexpresses Cyclin D1. Vega et al, in their review of 34 patients with lymphoma involving the pleura that was detected by pleural biopsy, found only 1 MCL among the 34 cases [16]. The most frequent type in their series was diffuse large B-cell lymphoma, followed by follicular lymphoma. The histologic pattern of MCL may be diffuse, nodular, or mantle zone, or a combination of the three patterns of growth. Some reports indicate a better prognosis for cases with a mantle zone pattern. Despite the small size and bland appearance of these cells, there is often more mitotic activity than in other histologically low-grade lymphomas. Diffuse forms and those which show high mitotic rates (> 20 HPF in diffuse and > 10 HPF in follicular) have a worse prognosis.

We report a unique case of coexistence of lung adenocarcinoma and mantle cell lymphoma of the pleura. The patient was admitted to the hospital due to persistent air leak from chest tube after an episode of spontaneous secondary pneumothorax, with no other specific clinical signs, and both tumors were incidentally identified. Poor results of pulmonary function tests and the coexistence of mantle cell lymphoma precluded the patient from further surgical treatment (lobectomy) for his early lung adenocarcinoma.

## Conclusion

The therapeutic management of such a combination of tumors requires separate consideration of their biologic behavior, the performance status of each patient individually and the estimated morbidity related to surgery and/or chemo-radiotherapy. We should note that any tissue resected during any non-oncologic intrathoracic procedure should be collected separately and sent for pathologic examination, especially in older people. Any scar detected in the lung should also resected during an intrathoracic procedure performed for benign disease, if do not require a major operation and do not add significant risk for the patient.

## Abbreviations

SCLC: squamous cell lung cancer; NSCLC: non small cell lung cancer; MCL: mantle cell lymphoma; HBV: hepatitis B virus; CT: computed tomography; FISH: fluorescence in situ hybridization; HPF: high power field; MALT: mucosa-associated lymphoid tissue.

## Consent

Written informed consent was obtained from the patient for publication of this case report and any accompanying images. A copy of the written consent is available for review by the Editor-in-Chief of this journal.

## Competing interests

The authors declare that they have no competing interests.

## Authors' contributions

DH carried out pathology examination, immunohistochemistry and drafted the manuscript. MB carried out immunohistochemistry and FISH assays and drafted the manuscript. Georgia Karayannopoulou carried out pathology examination, immunohistochemistry and FISH assays, has drafted the manuscript and has contributed to the interpretation of the results. Georgios Karkavelas has given final approval of the version to be published. GTK has assisted the surgeon, did the collection of the data and have been involved in the design and drafting of the manuscript. CNF has performed the operation and has critically revised the initial draft. IK carried out pathology examination, immunohistochemistry and FISH assays, has critically revised the initial draft and has given final approval of the version to be published
